# Fractures of the humeral shaft caused by arm wrestling: a systematic review

**DOI:** 10.1016/j.xrrt.2022.05.005

**Published:** 2022-06-22

**Authors:** Kiyohisa Ogawa, Atsushi Yoshida, Noboru Matsumura, Wataru Inokuchi

**Affiliations:** a Department of Orthopedic Surgery, Eiju General Hospital, Tokyo, Japan; b Department of Orthopedic Surgery, National Hospital Organization Saitama Hospital, Saitama, Japan; c Department of Orthopedic Surgery, School of Medicine, Keio University, Tokyo, Japan

**Keywords:** Arm wrestling, Adult, Humeral fracture, Eccentric muscle contraction

## Abstract

**Background:**

Arm wrestling is a popular sport/game that may result in various injuries. The most common arm wrestling injury in adults is humeral shaft fracture. This study aimed to elucidate the current understanding of humeral shaft fracture caused by arm wrestling and propose the possible mechanism.

**Methods:**

The PubMed and Web of Science databases were searched using the terms “arm wrestling” and “humeral fracture” as well as “sports” and “humeral fracture” in accordance with the Preferred Reporting Items for Systematic Reviews and Meta-Analyses guidelines. The inclusion criteria were English full-text articles and notable full-text articles in other languages concerning humeral shaft fracture caused by arm wrestling that described the patients' characteristics and presented adequate images or a detailed description of the fracture to confirm the fracture details. The exclusion criterion was a lack of appropriate images or detailed description of the fracture. Fifty-seven studies were identified. The patients' demographics were evaluated. The details of fractures, primary radial nerve palsy, match status, provided fracture treatment, and outcomes were evaluated using the chi-squared test. The relationship between fracture site and the patient's age was analyzed using Student's *t*-test.

**Results:**

One hundred fifty-three patients, 82% of whom were males aged 15-34 years, were identified. With only a few exceptions, almost all patients were injured in recreational matches. The injured limb was the right arm in 65% of patients (n = 141). The patient's physical characteristics, the opponent's physical characteristics compared with those of the patient, and the match status at the time of injury varied between cases. Among the 46 patients with known match details, all were injured when one of the wrestling opponents suddenly added more force in an attempt to change the match status. The fracture configuration was spiral in all cases, and 48% of fractures had an associated medial butterfly fragment. The fracture site was the distal third or the junction between the distal and middle thirds in 90% of cases. Although primary radial nerve palsy was recognized in 19 of 103 patients (18.4%), all resolved spontaneously.

**Conclusion:**

Although humeral shaft fracture caused by arm wrestling occurred mostly in male players aged 15-34 years, this injury may affect any player regardless of the match status, player's and opponent's physical characteristics, and age. The direct cause is torsional force generated by the internal rotators. A sudden change from concentric to eccentric contraction of the internal rotators is likely to cause fracture.

Arm wrestling is a popular sport and recreational game practiced worldwide by both men and women of all ages. Although the most common injury to adults is a humeral shaft fracture, arm wrestling can also cause other serious injuries, such as scapular neck fracture,^15^ rupture of the subscapularis,^9^^,^^25^^,^^57^ rupture of the medial collateral ligament of the elbow,^44^ anterior dislocation of the elbow with or without olecranon fracture,^69^^,^^73^ olecranon fracture,^64^ anterior dislocation or fracture-dislocation of the radial head,^17^^,^^60^ radial shaft fracture or neck fracture,^14^^,^^15^^,^^23^ recurrent subluxation of the extensor carpi ulnaris tendon,^10^ triangular fibrocartilage complex injury,^58^ rupture of the ulnar collateral ligament of the thumb,^24^ and proximal phalanx fracture^21^; moreover, epiphyseal fracture-separation of the medial humeral epicondyle may occur in teenagers.^2^^,^^53^^,^^59^^,^^75^^,^^83^

The purpose of this review is to systematically evaluate the available literature to clarify the current understanding of humeral shaft fracture secondary to arm wrestling and propose the possible mechanism based on clinical and experimental evidence.

## Materials and methods

**Figure 1:**
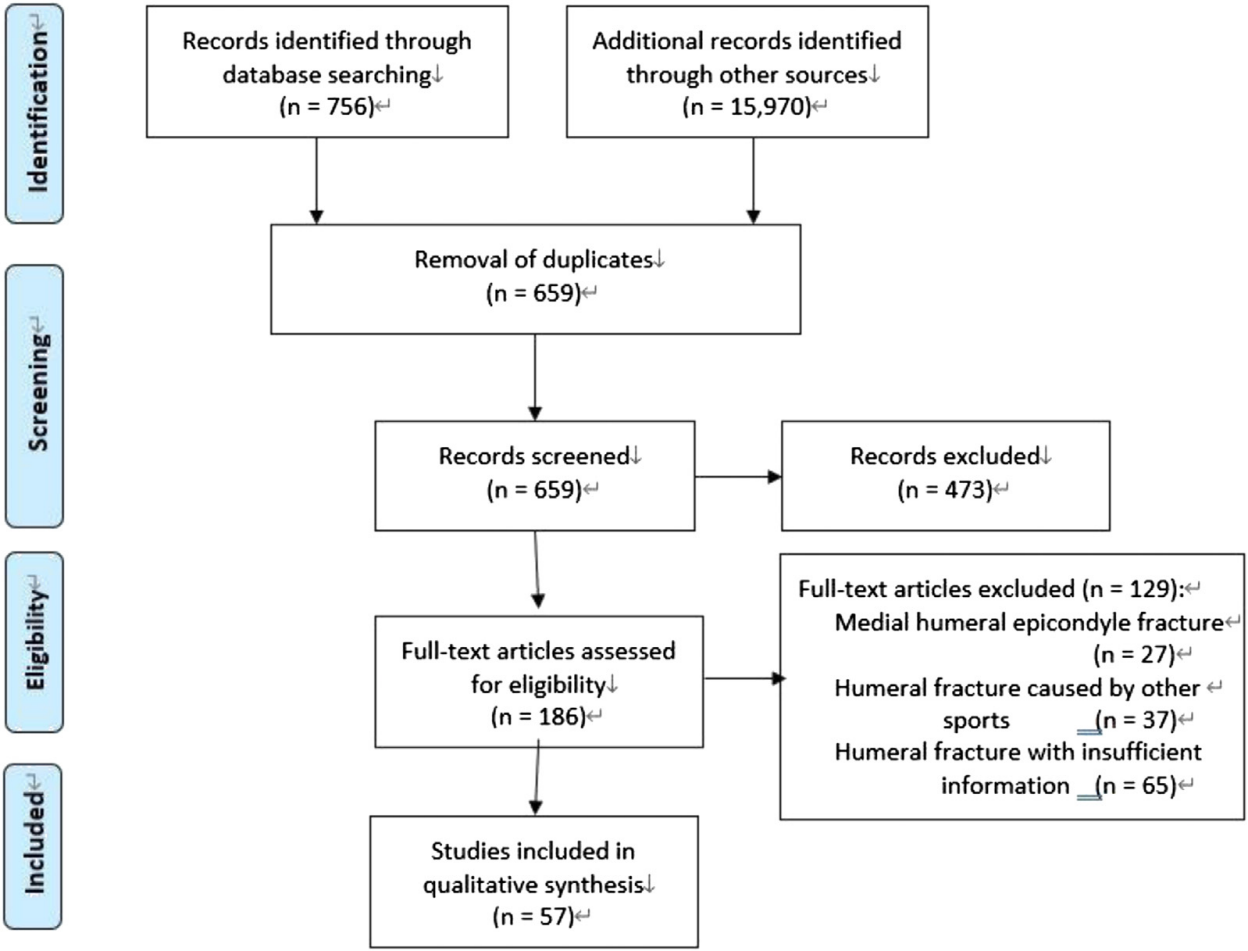
PRISMA flowchart of this study.

An electronic literature search was conducted following the Preferred Reporting Items for Systematic Reviews and Meta-Analyses guidelines using a checklist for systematic reviews.^51^ We also used the tool suggested by Murad et al^55^ to assess the methodological quality and synthesis of the case reports and case series. Although there were 8 questions in total, questions 5-7 were omitted because they were not relevant to this review (the possible maximum score was 5). The literature search was performed from March to May 2021, and the publication years of the included articles ranged from 1900 to 2020. The PubMed and Web of Science databases were searched using the terms “arm wrestling” and “humeral fracture” as well as “sports” and “humeral fracture” to identify relevant studies. Two reviewers (K.O. and N.M.) independently conducted the search and review. The inclusion criteria were English full-text articles concerning humeral shaft fracture caused by arm wrestling that described the patients' characteristics and presented the appropriate images or description of the injury situation using widely accepted classification methods to confirm the details of the humeral shaft fracture. The exclusion criteria were descriptive articles or cases without appropriate images or detailed description of the fracture to enable evaluation of the injury details, case series in which the characteristics of individual patients were not clearly described, and articles with scores of ≤4 using Murad's assessment tool.^55^ Citation tracking was carefully conducted to find additional related articles written in English and notable full-text articles written in other languages, which were selected and added to the qualitative synthesis. The article selection process is shown in Figure 1.

Because of the relative infrequency of these injuries, almost all studies were case reports or small case series including fewer than 10 cases; the exception was 7 case series that included 10 or more cases.^12^^,^^27^^,^^39^^,^^45^^,^^59^^,^^71^^,^^82^ Of these 7 case series, 2 that described the characteristics of individual patients were included in the analysis.^59^^,^^82^ Each patient was reviewed with respect to age, sex, job or physique, injured side, dominant arm, match status/details, posture in the match, opponent's physical characteristics, fracture configuration/site, associated injuries, type of treatment, and outcome. Although the classification of the humeral shaft divided into 3 equal parts was used for the determination of the fracture site and was essentially based on the judgment of the reported authors, it was modified by the images provided in a few reports. In some reports that used the classification in which the humeral shaft is divided into 5 or 10 equal parts,^59^^,^^82^ we converted the classification to that of 3 equal divisions. The 2 reviewers discussed the choice of papers and the cases to be adopted, and they ultimately reached agreement regarding all papers and cases. Ultimately, 57 studies were included in the analysis.

The items selected for comparison using the chi-squared test were the fracture site, presence or absence of a third fractured fragment and primary radial nerve palsy, match status, and fracture treatment (nonoperative or operative methods). The relationship between the fracture site (excluding fractures of the proximal part of the diaphysis) and the patient's age was analyzed using Student's *t*-test. The relationship between the reported year and fracture treatment was analyzed using the Mann–Whitney U-test. The level of significance was set at <.05.

## Results

**Figure 2:**
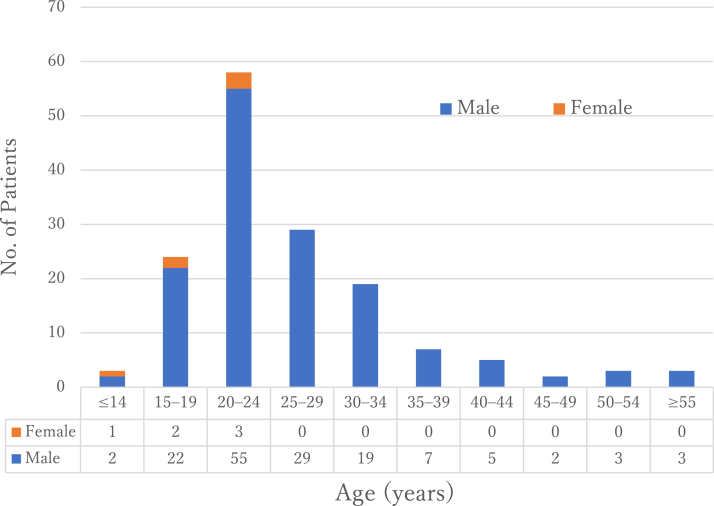
Age distribution and sex of patients with humeral shaft fracture caused by arm wrestling. Although the age distribution was wide, the peak age group was 20-24 years.

The 57 included articles described a total of 153 patients with a humeral shaft fracture. Of the 153 patients, 147 (96%) were male and 6 (4%) were female. The patients' mean age was 26.1 ± 9.0 years (range, 10–63 years). All 6 female patients were aged ≤24 years (range, 10–24 years).^2^^,^^26^^,^^30^^,^^32^^,^^59^ Male patients aged 15-34 years accounted for 82% (125/153) of the patients (Fig. 2).

All patients incurred a sudden-onset humeral shaft fracture during arm wrestling with no antecedent symptoms. Most patients were injured in a recreational arm wrestling match with their friends or colleagues, except for a few patients who were injured in an official match.^32^^,^^59^ Among 54 adult patients whose body mass index was stated, 40 had a body mass index within the normal range (18.50-24.95 kg/m^2^), 13 had mild thinness (17.00-18.49 kg/m^2^) or preobesity (25.00-29.99 kg/m^2^), and 1 had class II obesity (35.00-39.99 kg/m^2^) according to the criteria of the World Health Organization.^4^^,^^19^^,^^42^^,^^59^^,^^82^^,^^83^^,^^85^ Among the 28 patients whose physical characteristics were mentioned, 24 patients were muscular and 4 were of medium build.^8^^,^^13^^,^^18^^,^^37^^,^^40^^,^^47^^,^^54^^,^^61^^,^^85^, ^86^, ^87^ Among the 51 patients whose participation in regular sports activities was reported, 33 were engaged in regular sports activities.^8^^,^^19^^,^^47^^,^^59^^,^^82^^,^^85^ Among the 50 patients for whom alcohol consumption at the time of injury was mentioned, 27 (54%) patients had consumed alcohol.^18^^,^^19^^,^^37^^,^^42^^,^^47^^,^^59^^,^^63^^,^^80^^,^^81^ The injured limb was the right arm in 92 patients and the left in 49 patients (n = 141). The injured side was the dominant side in 51 (59%) patients and nondominant side in 35 (41%) patients (n = 86).^7^^,^^18^^,^^19^^,^^32^^,^^38^^,^^42^^,^^47^^,^^59^^,^^67^^,^^75^^,^^80^, ^81^, ^82^, ^83^^,^^85^^,^^86^ The posture during the match was prone for 1 patient, sitting for 26, and half-rising or standing for 22 (n = 49).^38^^,^^59^^,^^82^^,^^83^^,^^86^ Based on the patients' statements, the opponent was physically characterized as smaller/weaker than the patient in 4 cases, similar to the patient in 32, and larger/stronger than the patient in 15 (n = 51).^8^^,^^13^^,^^18^^,^^37^^,^^56^^,^^59^^,^^63^^,^^82^^,^^85^ The match status at the time of injury was in the winning phase for 14 patients, even for 37, and in the losing phase for 33 (n = 84).^1^^,^^4^^,^^6^^,^^13^^,^^16^^,^^18^^,^^26^^,^^30^^,^^32^^,^^37^^,^^38^^,^^40^^,^^47^^,^^48^^,^^54^^,^^59^^,^^61^^,^^75^^,^^77^^,^^80^^,^^82^^,^^83^^,^^85^ The details of the match situation at the time of injury were described for 46 of the 84 patients whose match status was known. All 46 patients incurred the injury when one of the pair suddenly added more force in an attempt to end the match or to change the match status. The match status was in the winning phase for 9 patients, even for 17, and in the losing phase for 20.

**Figure 3:**
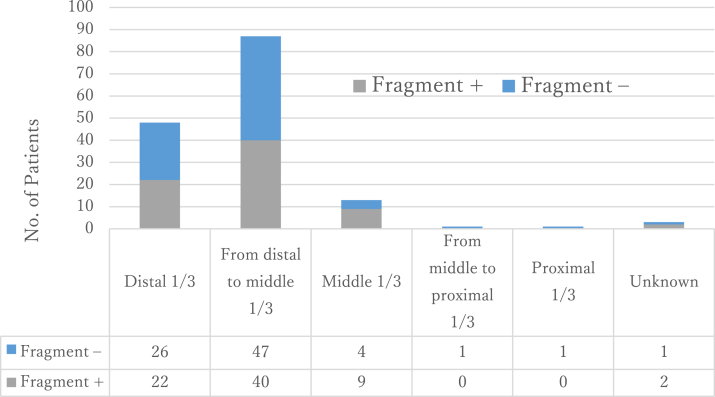
Prevalence of medial butterfly fragment. Approximately half of the patients had a quadrilateral third fragment on the medial side of the fracture site.

The fracture configuration was spiral in all patients (n = 153). The fracture site was the distal third in 48 patients, the junction between the distal and middle thirds in 87, the middle third in 13, the junction between the middle and proximal thirds in 1, the proximal third in 1, and unknown in 3 (n = 153). Almost all fractures were closed with the exception of one type 1 open fracture according to the Gustilo classification.^19^ A third fragment (called a butterfly fragment) on the medial side of the fracture was present in 73 (48%) of 153 patients (Fig. 3). In accordance with the AO/OTA classification, 80 patients had 12A1 fractures and 73 had 12B1 fractures. The fracture site was not correlated with the match status (*P* = .4727; n = 84). The presence of a third fragment was not correlated with the main fracture site (*P* = .354; n = 150), match status (*P* = .2513; n = 84), or patient age (*P* = .4726; n = 153).

Although the presence or absence of a neurovascular disorder was mentioned for only 103 patients, primary radial nerve palsy was the only concurrent injury in 19 (18.4%) patients,^19^^,^^40^^,^^42^^,^^56^^,^^59^^,^^80^^,^^81^ whereas secondary radial nerve palsy that occurred during or after treatment was recognized in another 5 patients.^13^^,^^19^^,^^40^ There was no significant association between radial nerve palsy and the presence of a third fracture fragment (*P* = .3450; n = 103; Table I). There was no significant difference in the incidence of radial nerve palsy according to the fracture site (*P* = .3397; n = 100).

**Table I:** Relationships among radial nerve palsy, fracture site, and presence of a third fragment (n = 103).

Fracture site	Fragment+		Fragment−		Total	
	RNP+	RNP−	RNP+	RNP−	RNP+	RNP−
Distal 1/3	3	14	1	17	4	31
Between distal and middle 1/3	3	21	10	24	13	45
Middle 1/3	1	4	1	1	2	5
Between middle and proximal 1/3	0	0	0	1	0	1
Proximal 1/3	0	0	0	0	0	0
Unknown	0	2	0	0	0	2
	7	41	12	43	19	84

*RNP*, radial nerve palsy.

The treatment method was nonoperative in 59 patients (mainly application of a hanging cast and/or functional bracing), operative in 80, and unknown in 14 (n = 153). The fixation device used for operative treatment was a plate in 59 patients, screws in 9, intramedullary nails in 8, and other devices in 4 (n = 80). The type of fracture treatment was unrelated to the presence of a third fragment (*P* = .7669; n = 139), but operative treatment was more common in patients with primary radial nerve palsy (*P* = .0175; n = 91). In addition, based on the publication year, operative treatment was more common than nonoperative treatment in more recent publications (*P* < .0001; n = 139); in particular, the incidence of plate fixation increased sharply in recent cases (Table II). Thirty patients (10 treated nonoperatively and 20 treated operatively) were followed up for 6 months or longer after injury (mean, 15.3 ± 12.5 months; n = 30). Although only one study used a widely accepted evaluation method (Mayo Elbow Performance Score),^19^ the reported treatment outcome was excellent, with no residual symptoms, limitation of range of motion, or elbow instability that impaired daily activities or sports activity in 26 patients. For the other 4 patients treated nonoperatively, the treatment outcome was rated as good (defined as the presence of ≤15° extension loss of the elbow and limitation of shoulder range of motion that does not impair daily or sports activities): 3 patients had 10°-15° extension loss of the elbow,^59^ and one patient had limited shoulder abduction.^80^ The primary radial nerve palsy observed in 19 patients (5 treated nonoperatively and 14 treated operatively) resolved spontaneously without surgical intervention to the nerve or residual disability. The secondary radial nerve palsy that occurred during treatment in 5 patients fully recovered without any additional treatment. With the exception of the previously mentioned cases of secondary radial nerve palsy, there were no reports of intraoperative, postoperative, or late complications such as tardy radial neuropathy.

**Table II:** Selected treatment method over time.

Publication year	Nonoperative treatment	Operative treatment		Unknown
		Plate	Other devices	
1953-1960	4	1	1	6
1961-1980	18	0	4	3
1981-2000	32	19	9	2
2001-2020	5	39	7	3
Total	59	59	21	14

## Discussion

Humeral shaft fractures constitute 1%-2% of all fractures and 13%-27% of all humeral fractures.^29^^,^^79^ The incidence of humeral shaft fracture is 14.5/100,000 per year among people aged ≥16 years.^22^ Male patients comprise more than half (55%-63%) of the patients with humeral shaft fracture, especially male patients aged 10-30 years.^22^^,^^66^^,^^74^^,^^79^ Sports-related injury accounts for 5%-7% of humeral shaft fractures.^22^^,^^29^^,^^74^ However, no data are available regarding the incidence of humeral shaft fracture related to arm wrestling.

Although it is unclear when the first scientific paper on humeral shaft fractures due to arm wrestling was published, 23 reported cases of humeral shaft fracture caused by arm wrestling had been published by 1905.^5^ Among these early cases, the autopsy of a 24-year-old man who incurred a humeral shaft fracture due to arm wrestling and died of pneumonia 8 days after the injury revealed a spiral fracture with a medial third fragment.^52^ The first article on humeral shaft fracture with images was presented in 1953.^26^ Humeral shaft fracture due to arm wrestling is truly an international phenomenon that has been reported either with or without images in Europe,^1^^,^^2^^,^^7^^,^^8^^,^^12^^,^^16^^,^^19^^,^^26^^,^^37^^,^^42^^,^^47^^,^^48^^,^^56^^,^^61^^,^^63^^,^^67^^,^^86^^,^^87^ Asia,^4^^,^^13^^,^^18^^,^^30^^,^^38^^,^^40^^,^^44^^,^^53^^,^^59^^,^^74^^,^^81^, ^82^, ^83^, ^84^, ^85^ North and South America,^6^^,^^32^^,^^77^^,^^80^ Morocco, and New Zealand. Considering that humeral shaft fractures due to arm wrestling have been reported in many countries and that arm wrestling has been widely performed worldwide for many years, numerous cases are likely to have gone unreported.

**Figure 4:**
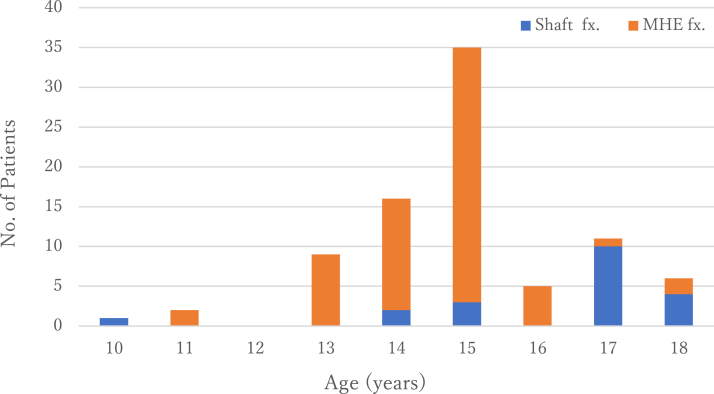
Incidences of humeral shaft and medial humeral epicondylar fractures in patients aged 10-18 years. In those aged 14-18 years, arm wrestling caused both humeral shaft and medial humeral epicondylar fractures. *fx*., fracture; *MHE*, medial humeral epicondylar.

In the present study, most patients were male (96%, 147/153). The patients' age ranged widely from 10 to 63 years, with most patients in their early 20s. Other than one 10-year-old girl with obstetric paralysis,^2^ the youngest patients were two 14-year-old boys.^6^^,^^75^ Avulsion fractures of the medial humeral epicondyle due to arm wrestling occurred in boys aged 11-18 years as far as we investigated, which suggests that arm wrestling is likely to result in both humeral shaft and medial humeral epicondyle fractures in those aged 14-18 years (Fig. 4).

The morphology of traumatic humeral shaft fracture was simple (OTA type A) in 56%-68% of cases,^22^^,^^66^^,^^74^ includes a third fragment (OTA type B) in 26%-34%, and was comminuted in 9%-10%.^22^^,^^66^ The fracture configuration was spiral in 11%-30% of cases.^33^^,^^62^^,^^70^^,^^79^ In the present review, the fracture configuration was spiral in all cases; this differs from the configuration of overall traumatic humeral shaft fractures. In the present study, the prevalence of a third fragment (butterfly fragment) on the medial side of the fracture site was about 50%, which is higher than the incidence reported for overall traumatic humeral shaft fractures.^22^^,^^66^ Although a previous study suggested that the position of the arm during the match appeared to be critical in determining the fracture pattern and location,^80^ there was no relationship between the match status and the fracture pattern or site in the present study.

In the present study, humeral shaft fractures due to arm wrestling mainly occurred in the distal third and at the junction between the distal and middle thirds, accounting for 90% (135/150). Two patients with fractures in the proximal third and at the junction between the proximal and middle thirds had no common distinctive characteristics.^59^^,^^83^ Previous studies have shown that the traumatic fracture site of the humeral shaft is the proximal third in 15%-25% of cases, middle third in 49%-68%, and distal third in 11%-35%.^66^^,^^74^ However, one study showed that the fracture site was the proximal third in 40.8%, middle third in 43.2%, and distal third in 16.0%.^22^ Therefore, fractures caused by arm wrestling tend to be more distal than those in the general population.

A 5.9- to 6.3-cm segment of the radial nerve is in direct contact with the posterior humerus from 17.1-15.7 cm to 10.9 cm proximal to the lateral humeral epicondyle with or without a structural groove in the humerus.^3^^,^^11^ On entering the anterior compartment of the upper arm, the radial nerve has very little mobility.^11^ These previously mentioned areas in the middle third and at the junction between the middle and distal thirds of the humeral shaft are considered the sites at greatest risk of injury. Therefore, although the overall prevalence of radial nerve palsy after humeral shaft fracture is 9.2%-12.2%,^31^^,^^33^^,^^62^^,^^70^ fractures of the middle third and the junction between the middle and distal thirds had a significantly greater association with radial nerve palsy than fractures in other parts (0%-2% of fractures in the proximal third, 7.5%-15.2% of fractures in the middle third, and 13%-23% of fractures in the distal third).^22^^,^^33^^,^^66^^,^^70^^,^^74^^,^^79^ Regarding the fracture configuration, radial nerve palsy is more likely in patients with transverse and spiral fractures than in those with oblique and comminuted fractures.^70^ In the present review, the incidence of primary radial nerve palsy was 18.4% (19/103), which is higher than the incidence of primary radial nerve palsy in overall traumatic humeral shaft fractures; this may be because all but 5 cases (including 3 unknown cases) of humeral shaft fracture due to arm wrestling comprised a spiral fracture in the middle third or more distal parts. Radial nerve injury associated with a closed humeral shaft fracture has a high rate of spontaneous resolution, reportedly ranging from 60% to 96%.^31^^,^^46^^,^^62^^,^^70^ In the present review, all cases of primary radial nerve palsy observed in 19 patients resolved spontaneously without the need for surgical intervention to the nerve. Therefore, careful observation is the first choice of treatment for radial nerve palsy associated with humeral shaft fractures due to arm wrestling.

Arm wrestling is a sport in which 2 opponents face each other with their bent elbows placed on a table and hands firmly gripped; each player then attempts to force the opponent’s hand down onto the table top. In arm wrestling, an internal rotational force is applied to the proximal humerus by the internal rotators of the shoulder, such as the pectoralis major, subscapularis, teres major, latissimus dorsi, and deltoid muscles.^34^^,^^59^^,^^72^ In contrast, the force applied to the hand by the opponent acts as an external rotational force via the forearm in the distal humerus.^34^^,^^72^ An experimental study demonstrated a large amount of muscle activity in the predominant phase in the flexor carpi ulnaris, pectoralis major, and latissimus dorsi.^34^ Furthermore, the normalized root mean square values of the pectoralis major muscle significantly increase from the initial position to the winning position, whereas other muscles show decreasing or constant values.^72^ A torsional force is subsequently applied to the humeral shaft by the internal rotation force acting on the proximal humerus and the external rotation force acting on the distal part of the humerus.

The material strength of bone depends on the loading direction. The shear strength of cortical bone (determined by torsion tests about the longitudinal axis and reflecting shear stress along the transverse and longitudinal planes) is about one-third the compressive strength,^36^ reportedly ranging from 68 to 70 MPa.^76^ According to experimental studies using the whole humerus, the mean torque required to initiate humeral shaft fracture ranges from 51 to 70 N·m, which is less than half the mean moment required to cause failure in bending.^41^^,^^68^^,^^78^^,^^84^ An experimental study using a constructed virtual model of the upper limb demonstrated that maximum bone stress resulting from the torsional loading that occurs during arm wrestling amounts to 60 MPa.^43^ Although some investigators performed morphological studies, the structure of the humerus explains the fracture location but not the mechanism of injury.^43^^,^^49^^,^^50^^,^^65^ In summary, there is no clear biomechanical evidence regarding whether arm wrestling causes humeral shaft fracture under normal conditions.

In the present study, although the number of analyzed cases was small, it is apparent that the patients' physical characteristics such as weight, height, and degree of muscle development as well as the opponent's physical characteristics compared with those of the patient varied, and injuries occurred even on the nondominant side, in older adults, and in females. Although some studies have indicated that humeral fracture due to arm wrestling seems to occur only during either the draw or losing phase,^54^^,^^80^ the present study revealed that humeral fracture occurred in any match status and any player. According to the mechanostat theory, mechanical loading results in bone structure that resists habitual loads, and the mechanical properties of bone (bone mass, geometry, and strength) adapt in accordance with the mechanical function required for everyday usage and needs.^28^ Because the mechanical properties of bone are influenced by the amount, distribution, and intrinsic properties of bone material present in the direction of loading,^78^ the previously mentioned variation in patient characteristics and the fact that many patients were amateur arm wrestlers are unsurprising. However, it is difficult to explain how humeral fracture is caused by the patient's own muscle power in accordance with this theory. In the present review, all 46 patients whose match details at the time of injury were known were injured when one of the pair suddenly added more force in an attempt to change the status of the match, regardless of their match status. All players were readily able to move their trunk because their known posture during the matches was sitting or half-rising.^59^ One experimental study showed that an inclination or tilting of the attacker's trunk occurred during attacking.^34^ We consider that the shifting body weight causes an abrupt shift of the internal rotators from maximum concentric contraction to eccentric contraction when exerting maximum force. In addition, the force of the reflective counterattack from the opponent that is inevitably added to the attacker's hand results in a rapid change from concentric to eccentric contraction of the internal rotators. Studies have demonstrated that isokinetic eccentric strength is 20%-60% greater than isokinetic concentric strength,^20^^,^^35^ although no data are available on the muscle groups involved in arm wrestling. Based on these clinical and experimental studies, we infer that a large nonphysiological force due to eccentric muscle contraction of the internal rotators causes humeral shaft fracture. Bending and compressive forces seem to be involved in addition to rotational force^53^^,^^80^^,^^86^ because of the existence of a third fragment in approximately half of the patients in the present review. However, how these forces are generated and work remains unclear.

Some researchers consider one cause of arm wrestling–induced fracture to be a dysfunction of physiological motor control due to alcohol consumption. In the present review, 27 (54%) of 50 patients whose alcohol consumption status was known had consumed alcohol.^19^^,^^37^^,^^42^^,^^45^^,^^47^^,^^63^^,^^81^ The fact that almost half of the injuries occurred without alcohol consumption indicates that inebriation is probably not a significant factor.^59^ Alcohol consumption and the occurrence of this type of fracture may reflect the habitual social culture of gathering in pubs and bars; however, there seems to be no direct or indirect causal relationship. Although some researchers have stated that arm wrestling–induced fractures can result from violent uncoordinated muscular action, there is no scientific evidence to support this.^53^^,^^54^^,^^84^ Because no patients had antecedent pain in the injured arm, there was no possibility of stress fracture.^59^

The present review had several limitations. First, because the number of cases that met the inclusion criteria was relatively small, reports with a medium risk of bias were included to maintain a sufficient number of cases for analysis. Second, most studies were case reports or retrospective case series with small numbers of patients. Third, because some studies did not report the patients' characteristics, medical history, or treatment method, the number of cases that could be analyzed differed for each analyzed item. Fourth, the variability in the reported outcome evaluation methods and small number of analyzed patients made it extremely difficult to perform meaningful comparisons between the outcomes of the different treatment methods. Finally, because only a small number of patients were followed up for a sufficient period, the occurrence of late complications could not be confirmed.

## Conclusion

Although humeral shaft fracture caused by arm wrestling occurred mostly in male players aged 15-34 years, such fracture can occur in any player regardless of the match status, player's and opponent's physical characteristics, and age. The fracture pattern tends to be more distal, with a slightly higher risk of radial nerve palsy in arm wrestlers than in the general population. Because all cases of primary radial nerve palsy resolved spontaneously, careful observation is the first-choice treatment for radial nerve palsy associated with humeral shaft fracture due to arm wrestling. The direct cause of injury was torsional force generated by the internal rotator muscles. A sudden change from concentric to eccentric contraction of the internal rotator muscles likely affects fracture occurrence.

## Acknowledgments

The authors thank Angela Morben, DVM, ELS, from Edanz (https://jp.edanz.com/ac) for editing a draft of this article.

## Disclaimers:

Funding: No funding was disclosed by the authors.

Conflicts of interest: The authors, their immediate families, and any research foundation with which they are affiliated have not received any financial payments or other benefits from any commercial entity related to the subject of this article.
